# Effects of husk and harvest time on carotenoid content and acceptability of roasted fresh cobs of orange maize hybrids

**DOI:** 10.1002/fsn3.179

**Published:** 2014-10-14

**Authors:** Oladeji E Alamu, Abebe Menkir, Bussie Maziya-Dixon, Olorunfemi Olaofe

**Affiliations:** 1 International Institute of Tropical Agriculture (IITA)P.M.B. 5320, Oyo Road, Ibadan, Oyo State, Nigeria; 2 Ekiti State UniversityAdo-Ekiti, P.M.B. 5363, Ekiti State, Nigeria

**Keywords:** Harvesting time, husk, orange maize, provitamin A, roasting, *β*-carotene

## Abstract

Vitamin A deficiency (VAD) is a major public health problem in many developing countries. Orange maize is preferred as green maize and consumed roasted on the cob, especially in Nigeria. This research work was to evaluate the effects of harvest time and husk on the carotenoid contents and sensory properties of roasted orange maize hybrids. The results showed that husk (roasting forms) and harvesting time had significant effects (*P* ≤ 0.001) on the carotenoids and the sensory properties. There was general increase in *β*-carotene and provitamin A (PVA) values as the harvesting time increases. The *β*-carotene and PVA values for roasted orange maize hybrids with husk were higher than those for roasted without husk. Hybrid 5 had the highest *β*-carotene concentration and PVA value at 27 days after pollination (DAP) and 34DAP when unprocessed and roasted without husk. This information can help researchers in choosing proper roasting methods to increase the retention of high levels of *β*-carotene and PVA in orange maize that can be delivered to consumers through nutrition education.

## Introduction

Vitamin A deficiency (VAD) is a major public health problem in many developing countries and is the most common cause of preventable blindness with estimated 250,000–500,000 vitamin A-deficient children going blind yearly (West 2003). In these countries, it is estimated that about two-thirds of child mortality can be prevented through public health interventions (Jones et al. 2003). VAD in humans is associated with susceptibility to infection, night blindness, rough and scaly skin, and diminished teeth and bone development (Lonzano-Alejo et al. 2007). Although VAD can be effectively addressed through supplementation programs, these interventions are costly and may be difficult to sustain. Food-based strategies, including biofortification of staple food crops with nutrients, can be used for VAD in developing countries (Gibson and Hotz 2001; Ruel 2001; Menkir et al. 2008). Maize is the most important cereal grain, accounting for 74% of the aggregate output (FAOSTAT 2011). It ranks third in the world production of food grains surpassed only by rice and wheat (FAOSTAT 2011). Maize would have added nutritional value if the grain contained appreciable level of carotenoids (Egesel et al. 2003). Efforts to develop maize with increased concentrations of provitamin A carotenoids generated some orange maize lines and hybrids with high levels of *β*-carotene (Egesel et al. 2004; Menkir and Maziya-Dixon 2004; Menkir et al. 2008). Studies found significant genetic variation in carotenoids in orange maize lines and hybrids adapted to temperate (Weber 1987; Kurilich and Juvik 1999; Egesel et al. 2003) and tropical environments (Menkir and Maziya-Dixon 2004; Menkir et al. 2008). The effectiveness of a biofortificaton strategy depends on how traditional processing and food preparations affect the nutritional content in products commonly consumed by the disadvantaged sector of the society.

Orange maize is preferred to green maize and consumed boiled or roasted on the cob to bridge the hunger gap after a long dry season. The roasted corn has gained popularity among Nigerians who see it as a good means of satisfying their hunger for the day. It appears that roasted corn has gradually moved from being an item of refreshment to becoming the mainstream Nigerian meals. Some studies on the effect of roasting on the nutrient content of white maize reported significant reduction in the levels of soluble solids, minerals and vitamins (Ayatse et al. 1983; Okoh 1998; Barampama and Simard 1995). Other studies on yellow maize found that cooking causes losses in provitamin A activity through isomer formation (Khachik et al. 1992). Results of studies on the effect of cooking on the retention of carotenoids found an increase in carotenoid content in some vegetables while in others a decrease in carotenoids was observed (Mosha et al. 1997). However, limited information is available regarding the effect of roasting on the carotenoid content in tropical orange maize. The objective of this study was therefore to evaluate the effects of harvest time and the retention or removal of husks on the carotenoid contents and sensory properties of roasted fresh cobs harvested from orange maize hybrids.

## Material and Experimental Methods

### Genetic material

Freshly harvested cobs from eight orange maize hybrids with varying carotenoid content and endosperm texture were used for this study. These hybrids were developed at IITA from diverse lines with high provitamin A (PVA) content. The viable seeds of eight selected orange maize hybrids were planted in two separate trials at Ibadan (7°22′N, 3°58′E, altitude 150 m) and Ikenne (10°40′N, 8°77′E, altitude 730 m) with different and known meteorological information, in early seasons of April to August 2010 and 2011. The hybrids were arranged in a randomized complete block design (RCBD) with three replications. Cobs of each hybrid were self pollinated to minimize contamination from other pollen sources.

### Field sampling

Plants were randomly prelabelled on the field for the three harvest maturity stages of 20, 27 and 34 days after pollination (DAP) (the day after pollination started from 50% anthesis or 50% silk emergence which was 57 days after planting) for each hybrid. They were harvested at 08.00 h on the relevant dates. A total of 20 selected cobs of each hybrid were harvested from each plot and these were pooled to give 60 cobs per hybrid per harvest. They were packed in mailing sacks and conveyed to the laboratory as soon as possible (Osanyintola et al. 1992). In the laboratory, each hybrid was divided into three sets for chemical assays, boiling with intact husk (undehusked cobs) and boiling without husk (dehusked cobs). All the selections and divisions were strictly randomised.

### Processing of freshly harvested orange maize

The 15 selected harvested cobs of each hybrid were roasted with intact husk and 15 selected cobs were dehusked and roasted on hot charcoal burning on wire gauze until the seeds were cooked and turned brown according to the local practice as described in other studies (Osanyintola et al. 1992). The roasting time varied with harvest times for both forms of roasting. Dehusked cobs from 20, 27 and 34DAP harvests roasted at 15, 12, and 10 min, respectively, while undehusked cobs from 20, 27 and 34DAP harvests roasted at 20, 15, and 10 min, respectively. All the harvested cobs were processed within 12 h after harvesting. The samples for sensory evaluation were kept warm in a cooler equipped with Styrofoam. The unprocessed and processed orange maize cobs for each hybrid and from each harvest meant for chemical assays were carefully shelled, uniformly freeze-dried using Labconco Freezone 4.5L (at temperature of −54°C and vacuum pressure of 0.45 mbar). The freeze-dried samples were milled using Laboratory mill 310 from PERTEN (Hägersten, Sweden) using sieve size 0.5 mm, packed in a dark sample polythene whirl-pack and stored at −80°C until analyzed for carotenoids.

### Evaluation of sensory properties

Sensory evaluation was carried out on the roasted fresh orange maize samples within 24 h after harvesting. The serving and experiment were performed under standard sensory test conditions (Larmond 1977). The samples were evaluated by 10 trained panels and degree of liking and attributes ratings were determined on a nine-point hedonic scale where 1 = dislike extremely and 9 = like extremely for color, aroma, chewiness, appearance, taste, and overall acceptability/likeness. The overall likeness ratings are means of duplicate averages of 10 panelists’ hedonic scores. The selected panelists were screened for ‘normal’ sensory acuity through taste, aroma and texture/chewiness identification tests. Basic taste recognition assessment was conducted using solution of sucrose, sodium chloride, citric acid and quinine sulfate. Aroma and texture recognition tests were done following the method recommended by Watts et al. (1989). Panelists started with the selection of important quality attributes of boiled fresh maize followed by a technique of evaluation and the use of a standard rating scale. Panelists selected color, aroma, chewiness, appearance, and taste as the most important quality attributes of roasted maize. They were served with the roasted samples in duplicates while they were still warm to touch.

### RP-HPLC carotenoid analysis

The method of Howe and Tanumihardjo (2006) was employed to assess the samples for carotenoid composition and content. The extraction of carotenoid from dried maize (0.6 g) was done by adding ethanol (10 mL) containing 0.1% butylated hydroxyl toluene (BHT), using a vortex, mixer, and a 5 min ethanol precipitation in 85°C water bath. Potassium hydroxide (500 *μ*L, 80% w/v) was added to the mixture to saponify the interfering oil. Samples were vortexed and placed in a water bath (85°C) for 5 min. It was vortexed again and returned to the water bath for an additional 5 min. Upon removal they were immediately placed in an ice bath where 3 mL of cold deionized water was added. Carotenoids were separated three times with addition of 3 mL of hexane, vortexed, and then centrifuged (1200*g*) for 5 min. The combined hexane fractions were washed with deionized water three times, vortexed, and centrifuged for 5 min at 1200*g*. The hexane fractions were dried down using TurboVap LIV concentrator under nitrogen gas. The dried extract was reconstituted in methanol/dichloromethane (1 mL, 50:50 v/v) and 100 *μ*L aliquot were injected into the HPLC system for analyses of *α*-carotene, *β*-carotene (*cis and trans* isomers) and *β*-cryptoxanthin. Waters HPLC system (Water Corporation, Milford, MA) consisting of a guard-column, C30 YMC Carotenoid column (4.6 × 250 mm, 3 *μ*m), Waters 626 binary HPLC pump, 717 auto-sampler and a 2996 photodiode array detector (PDA) was used for carotenoids quantification. The system operated with Empower 1 software (Waters Corporation). Solvent A consisted of methanol: water (92:8 v/v) with 10 mmol/L ammonium acetate and solvent B consisted of 100% methyl tertiary-butyl ether. Gradient elution was performed at 1 mL/min with the following condition: 29 min linear gradient from 83% to 59% A, 6 min linear gradient from 59% to 30% A, 1 min hold at 30% A, 4 min linear gradient from 30% to 83% A and a 4 min hold 83%. *β*-carotene eluted at ˜25 min. Chromatograms were generated at 450 nm and identification of *α*-carotene, *β*-carotene (*cis and trans* isomers), and *β*-cryptoxanthin were determined using external standard method based on the calibration curve from pure standards and verification of absorption spectrum and co-elution with available authentic standards. Standards of *α*-carotene, *β*-carotene, and *β*-cryptoxanthin were purchased from CaroteNature, GmbH (Lupsingen, Switzerland). Solvents were HPLC grade.

### Statistical analysis

Analytical data were reported as mean ± standard deviation of at least duplicate independent extractions of samples from two locations and for two seasons. The provitamin A (PVA) content of the maize was calculated by adding the amount of *β*-carotene (*cis and trans* isomers) to one-half of the amounts of *α*-carotene and *β*-cryptoxanthin. On the basis of the molecular structure, *α*-carotene and *β*-cryptoxanthin are considered to have 50% of the provitamin A activity of *β*-carotene (Food and Nutrition Board of the Institute of Medicine 2001). Thus, the amount of provitamin A activity obtained from *α*-carotene and *β*-cryptoxanthin was calculated to be half of the amount obtained from *β*-carotene. The percent true retention (%TRT) for *β*-carotene and PVA was calculated using the method recommended and described by Murphy et al. (1975). The %TRT was found to give more accurate retention data for the carotenoid retention, taking into account changes in food weight during cooking. Data generated were subjected to analysis of variance (ANOVA) and descriptive statistics, using statistical analysis system (SAS) software package 9.2. (SAS Institute 2000). Least significant difference (LSD) test was used for mean comparison.

## Results

The results of analysis of variance (ANOVA) showed that hybrid, husk, and harvesting time (maturity) had significant effects (*P* ≤ 0.001) on all the carotenoids of roasted orange maize hybrid investigated (Table1). Hybrid × husk and hybrid × maturity interactions were not significant for almost all provitamin A carotenoids (pVACs) whereas the husk x maturity and hybrid × husk × maturity interactions were significant for pVACs, including provitamin A activity (PVA). The results of this study revealed that replication, environment, and hybrid interaction was found to represent a small fraction of the total variation in the concentrations of total *β*-carotene and PVA when compared with variation among husk and maturity.

**Table 1:** Mean squares from the analysis of variance for the carotenoid properties of roasted orange maize evaluated at two locations for 2 years

Source	DF	MS				
		*β*-cryptoxanthin	*α*-carotene	Trans *β*-carotene	Total *β*-carotene	Provitamin A
Hybrid	7	15.30***	0.57***	5.86***	12.90***	30.90***
Husk	1	84.00***	3.45***	37.10***	65.30***	167***
Harvest time	2	61.10***	2.77***	40.30***	102***	213***
Hybrid × husk	7	1.22	0.03	0.24	0.66	1.45
Hybrid × harvest time	14	0.59	0.03	0.25	0.66	1.22
Husk × harvest time	2	4.27**	0.22**	2.00***	4.17***	10.30***
Hybrid × husk × harvest time	125	1.32**	0.058**	0.56***	1.30***	2.92***
Error	188	0.88	0.04	0.22	0.54	1.38

**, ***Significant at *P* ≤ 0.05, *P* ≤ 0.01 and *P* ≤ 0.001, respectively. ns, not significant *P* > 0.05.

### Effect of roasting without husk and harvest time

The *β*-carotene and PVA contents of hybrids unprocessed and roasted without husk increased with delayed in harvesting time (Table2). The *β*-carotene and PVA concentrations of cobs roasted without husk were lower than those unprocessed. Mean concentrations of *β*-carotene of unprocessed orange maize hybrids showed that hybrid 1 had the highest *β*-carotene and provitamin A concentrations of 2.90 ± 1.58 *μ*g/g and 3.91 ± 2.08 *μ*g/g, respectively at 20DAP. Hybrid 5 had the highest *β*-carotene and provitamin A concentrations of 3.99 ± 0.498 *μ*g/g and 5.73 ± 0.691 *μ*g/g, respectively at 27DAP and 4.80 ± 1.89 *μ*g/g and 6.95 ± 2.56 *μ*g/g, respectively at 34DAP. Thus, hybrid 5 had the highest concentrations of *β*-carotene and PVA accumulations both at 27DAP and 34DAP among all hybrids evaluated at different harvesting time.

**Table 2:** Means of *β*-carotene and provitamin A contents of unprocessed and roasted orange maize hybrids harvested at different harvesting time at two locations for 2 years

Hybrid	Unprocessed			Roasted without husk			Roasted with husk		
	20D	27D	34D	20D	27D	34D	20D	27D	34D
1*β—carotene (μg/g dry weight)*									
1	2.90 ± 1.58	3.35 ± 0.72	4.25 ± 1.34	2.08 ± 0.74	3.13 ± 0.93	3.03 ± 0.84	2.45 ± 0.70	3.09 ± 0.69	4.84 ± 1.63
2	1.52 ± 0.50	2.19 ± 0.50	3.36 ± 1.28	1.37 ± 0.70	1.65 ± 0.54	2.67 ± 1.21	1.44 ± 0.49	3.14 ± 1.47	3.62 ± 1.01
3	1.74 ± 0.66	3.32 ± 0.67	4.02 ± 1.23	2.02 ± 1.40	2.24 ± 0.43	2.99 ± 0.91	2.06 ± 0.77	3.76 ± 1.94	5.05 ± 2.31
4	1.83 ± 0.56	3.10 ± 1.08	3.77 ± 1.11	2.13 ± 0.54	2.93 ± 1.48	3.47 ± 1.23	2.37 ± 0.53	3.98 ± 1.20	4.47 ± 0.88
5	2.55 ± 1.22	3.99 ± 0.49	4.80 ± 1.89	2.21 ± 0.74	3.76 ± 1.18	4.40 ± 0.74	3.01 ± 0.94	4.12 ± 1.18	4.72 ± 0.92
6	2.47 ± 0.72	3.63 ± 0.82	4.51 ± 1.52	1.63 ± 0.72	3.09 ± 1.50	3.84 ± 0.93	3.14 ± 0.96	3.59 ± 1.39	5.00 ± 1.72
7	2.32 ± 0.55	3.58 ± 0.99	4.24 ± 0.97	2.57 ± 0.65	3.19 ± 0.59	3.39 ± 0.57	2.86 ± 0.92	3.66 ± 1.50	5.26 ± 1.11
8	1.61 ± 0.89	2.39 ± 0.48	3.52 ± 1.30	1.14 ± 0.48	2.17 ± 0.98	2.72 ± 0.97	1.55 ± 0.36	3.44 ± 1.72	2.91 ± 0.93
Mean	2.12	3.19	4.06	1.90	2.77	3.31	2.36	3.60	4.48
LSD (0.05)	1.21	1.21	1.21	0.36	0.36	0.36	0.46	0.46	0.46
CV (%)	2.99	2.42	1.50	3.13	3.10	2.23	3.40	1.28	2.26
1*Provitamin A (μg/g dry weight)*									
1	3.91 ± 2.08	4.77 ± 0.98	6.26 ± 2.06	3.08 ± 1.01	4.56 ± 1.17	4.29 ± 1.19	3.63 ± 1.02	4.64 ± 1.13	7.40 ± 3.16
2	2.29 ± 0.84	3.19 ± 0.80	5.12 ± 2.20	2.12 ± 1.10	2.48 ± 0.72	3.94 ± 1.84	2.25 ± 0.81	4.58 ± 1.95	5.17 ± 1.37
3	2.53 ± 1.10	4.98 ± 1.22	6.07 ± 1.92	3.14 ± 2.26	3.36 ± 0.55	4.51 ± 1.42	3.17 ± 1.16	5.57 ± 2.63	7.54 ± 3.41
4	2.79 ± 0.95	4.61 ± 1.45	5.94 ± 1.89	3.44 ± 0.77	4.37 ± 1.80	5.33 ± 1.91	3.52 ± 0.70	5.99 ± 1.53	7.03 ± 1.25
5	3.69 ± 1.93	5.73 ± 0.69	6.95 ± 2.56	3.34 ± 1.09	5.39 ± 1.37	6.47 ± 1.23	4.73 ± 1.55	6.10 ± 1.59	7.02 ± 1.44
6	3.62 ± 1.15	5.18 ± 1.01	6.76 ± 2.42	2.46 ± 1.08	4.53 ± 1.95	5.68 ± 1.64	4.99 ± 1.49	5.32 ± 2.05	7.57 ± 2.39
7	3.63 ± 0.97	5.52 ± 1.58	6.72 ± 1.66	4.02 ± 1.01	4.91 ± 1.08	5.08 ± 1.12	4.82 ± 1.65	5.70 ± 2.45	8.27 ± 1.82
8	2.47 ± 1.40	3.59 ± 0.68	5.27 ± 1.77	1.75 ± 0.73	3.24 ± 1.41	4.14 ± 1.56	2.45 ± 0.65	5.10 ± 2.37	4.61 ± 1.12
Mean	3.12	4.70	6.14	2.92	4.11	4.93	3.70	5.38	6.83
LSD (0.05)	0.70	0.70	0.70	0.53	0.53	0.53	0.69	0.69	0.69
CV (%)	2.65	2.37	1.38	3.22	2.98	2.21	3.61	1.33	2.31

LSD, least significant difference.

1 Parameter mean value ± SD.

When the orange maize hybrid cobs were roasted without husk, it was observed that hybrids 1, 3, 4, 5, and 7 showed higher *β*-carotene and PVA values than the overall mean of 1.90 *μ*g/g for *β*-carotene and 2.92 *μ*g/g for PVA at 20DAP but hybrid 7 had the highest *β*-carotene concentration of 2.57 ± 0.653 *μ*g/g and PVA value 4.027 ± 1.01 *μ*g/g. Hybrids 1, 4, 5, 6 and 7 showed higher *β*-carotene and PVA values than the overall mean of 2.77 *μ*g/g for *β*-carotene and 4.11 *μ*g/g for PVA at 27DAP but hybrid 5 had the highest *β*-carotene concentration of 3.76 ± 1.18 *μ*g/g and PVA value of 5.39 ± 1.37 *μ*g/g. Hybrids 4, 5, 6, and 7 showed higher *β*-carotene and PVA value than the overall mean of 3.31 *μ*g/g for *β*-carotene and 4.93 *μ*g/g for PVA at 34DAP but hybrid 5 had the highest *β*-carotene concentration of 4.40 ± 0.745 *μ*g/g and PVA value of 6.47 ± 1.23 *μ*g/g. Thus, hybrid 5 emerged the best hybrid that could be roasted without husk at 27DAP and 34DAP with optimum *β*-carotene and PVA values.

The *cis* to *trans*- *β*-carotene ratio of roasted without husk was evaluated and there was an increase in the ratio of *cis* to *trans*- *β*-carotene at 20DAP (0.680) and 27DAP (0.694) before a decrease at 34DAP (0.657) (Table3). Hybrids 1, 4, 5, 6, and 7 showed lower *cis* to *trans*- *β*-carotene ratio than the overall mean ratio of 0.644. Hybrids 1, 4, 5, 6, and 7 showed lower *cis* to *trans*- *β*-carotene ratio than the overall mean ratio of 0.694 at 27DAP and hybrid 5 had the lowest ratio of 0.649. Hybrids 3, 5, and 6 showed lower *cis* to *trans*- *β*-carotene ratio than the overall mean ratio of 0.657 at 34DAP and hybrid 5 had the lowest ratio of 0.599 (Table3). Hybrid 1 at 20DAP and hybrid 5 at 27DAP, and 34DAP were the best hybrids that had higher *trans*- *β*-carotene because of their low *cis* to *trans*- *β*-carotene ratio when fresh cobs of orange maize hybrids were roasted without husk.

**Table 3:** *Cis-β-*carotene to *trans-β*-carotene ratio of roasted fresh orange maize hybrids with and without husk at two locations for 2 years

Hybrids	Roasted with husk			Roasted without husk		
	*cis:trans-β*carotene ratio			*cis:trans-β*carotene ratio		
	20DAP	27DAP	34DAP	20DAP	27DAP	34DAP
1	0.607	0.617	0.617	0.644	0.668	0.677
2	0.635	0.532	0.605	0.766	0.740	0.696
3	0.617	0.596	0.712	0.685	0.754	0.631
4	0.576	0.541	0.526	0.645	0.665	0.662
5	0.572	0.531	0.487	0.649	0.649	0.599
6	0.560	0.540	0.560	0.669	0.653	0.654
7	0.581	0.510	0.550	0.653	0.686	0.673
8	0.573	0.604	0.594	0.730	0.739	0.663
Mean	0.590	0.559	0.581	0.680	0.694	0.657
LSD (0.05)	0.602	0.602	0.602	0.709	0.709	0.709
CV (%)	4.45	7.21	11.7	6.64	6.22	4.56

LSD, least significant difference.

The results of the percentage true retention of total *β*-carotene (%TRT [TBC]) of roasted maize hybrids without husk showed a marginal increase in %TRT (TBC) as the harvesting time increases. Hybrid 4 at 20DAP, hybrid 5 at 27DAP, and hybrid 6 at 34DAP were the best hybrids with high %TRT (TBC) (Table4). There was general decrease in %TRT of PVA as the harvesting time increases when fresh cobs of orange maize hybrids were roasted without husk. Hybrids 3 at 20DAP, hybrid 1 at 27DAP, and hybrid 5 at 34DAP emerged as the best hybrids with high %TRT of PVA and suggesting that roasting of fresh cobs orange maize hybrids without husk for higher retention of PVA is determined by hybrid and harvesting time (Table4).

**Table 4:** Percentage true retention (%TRT) of total *β*- carotene (TBC) and Provitamin A (PVA) of roasted fresh yellow hybrid maize with and without husk at two locations for 2 years

Hybrids	Roasted with husk			Roasted without husk		
	20DAP	27DAP	34DAP	20DAP	27DAP	34DAP
	%TRT (TBC)			%TRT (TBC)		
	
1	84.6	81.7	129	71.7	82.9	80.7
2	59.4	135	81.3	56.5	71.2	59.8
3	64.8	124	163	63.5	74.0	99.7
4	160	103	135	144	75.8	105
5	121	139	106	88.7	126	98.6
6	116	90.2	199	60.0	77.8	152
7	142	77.6	181	128	67.7	116
8	59.8	134	82.8	44.2	84.3	77.4
Mean	101	111	135	82.0	82.5	98.6
LSD (0.05)	2.43	2.43	2.43	0.902	0.902	0.902
CV (%)	38.9	23.0	32.6	43.7	22.5	28.2

	%TRT (PVA)			%TRT (PVA)		
1	92.8	97.3	118	78.7	95.6	68.5
2	98.3	144	101	92.8	77.9	76.9
3	125	112	120	124	67.5	74.3
4	126	130	118	123	94.8	89.8
5	128	107	101	90.4	94.2	93.0
6	138	103	113	67.9	87.4	84.0
7	133	103	123	111	88.8	75.6
8	99.4	142	87.5	70.7	90.2	78.5
Means	118	117	110	94.8	87.0	80.1
LSD (0.05)	1.90	1.90	1.90	0.750	0.750	0.750
CV (%)	15.1	15.9	11.3	23.6	11.2	10.3

LSD, least significant difference.

### Effect of roasting with husk and harvest time

There was also a general increase in *β*-carotene and PVA values as the harvesting time increases as found in roasted maize hybrids without husk (Table2). Hybrids 1, 4, 6, and 7 showed a higher *β*-carotene content than the overall mean of 2.36 *μ*g/g and hybrids 5, 6, and 7 showed higher PVA value than the overall mean of 3.70 *μ*g/g at 20DAP. Hybrid 6 had the highest *β*-carotene concentration of 3.14 ± 0.967 *μ*g/g and PVA value of 4.99 ± 1.49 *μ*g/g. Hybrids 3, 4, 5, and 7 showed higher *β*-carotene concentration and PVA value than the overall mean of 3.60 *μ*g/g for *β*-carotene, and 5.38 *μ*g/g for PVA at 27DAP. Hybrid 5 had the highest *β*-carotene concentration of 4.12 ± 1.18 *μ*g/g and PVA value of 6.10 ± 1.59 *μ*g/g. Hybrids 1, 3, 5, 6, and 7 showed higher *β*-carotene than the overall mean of 4.48 *μ*g/g and hybrids 1, 3, 4, 5, 6, and 7 showed higher PVA value than the overall mean of 6.83 *μ*g/g at 34DAP. Hybrid 7 had the highest *β*-carotene concentration of 5.26 ± 1.10 *μ*g/g and PVA value of 8.27 ± 1.8 *μ*g/g. Hybrid 7 at 34DAP emerged as the best with the highest values of *β*-carotene and PVA.

From the results of *cis*- *β*-carotene to *trans*- *β*-carotene ratio of roasted fresh orange maize hybrids with husk (Table3), hybrids 4, 5, 6, and 7 showed lower *cis* to *trans*- *β*-carotene than the overall mean ration of 0.590 at 20DAP and hybrid 6 had the lowest ratio of 0.560. Hybrids 2, 4, 5, 6, and 7 showed lower *cis* to *trans*- *β*-carotene ratio than the overall mean ratio of 0.559 at 27DAP and hybrid 7 had the lowest ratio of 0.510. Hybrids 4, 5, and 6 showed lower *cis* to *trans*- *β*-carotene ratio than the overall mean ratio of 0.581 at 34DAP and hybrid 5 had the lowest ratio of 0.487. Hybrid 6 at 20DAP, hybrid 7 at 27DAP, and hybrid 5 at 34DAP were the best hybrids because of their low *cis* to *trans*- *β*-carotene ratio. There was a decrease in the ratio of *cis* to *trans*- *β*-carotene at 20DAP (0.590) and 27DAP (0.559) before a marginal increase at 34DAP (0.581). This suggested a maturity effect on the isomers of *β*-carotene when fresh cobs of orange maize hybrids were roasted with husk. Here 27DAP had the lowest *cis* to *trans*- *β*-carotene ratio of 0.559 while hybrid 5 had the lowest ratio of 0.487 at 34DAP.

The results of the percentage true retention of total *β*-carotene (%TRT [TBC]) for roasted cobs of fresh orange maize hybrids with husk showed that hybrid 4 at 20DAP and hybrid 5 at 34DAP were the best hybrids with high %TRT (TBC) and higher than the control hybrid 8. Hybrid 5 not only had high %TRT (TBC) but as earlier shown low *cis* to *trans*- *β*-carotene ratio at both 27DAP and 34DAP, suggesting that this hybrid is the best when fresh orange maize hybrids were roasted with husk There was a marginal increase in %TRT (TBC) as the harvesting time increases when fresh orange maize hybrids were roasted with husk (Table4). The results of the percentage true retention of provitamin A (%TRT [PVA]) for roasted fresh orange maize hybrids with husk showed that hybrid 6 at 20DAP, hybrid 2 at 27DAP, and hybrid 7 at 34DAP were the best hybrids with high %TRT of PVA and suggesting that roasting of fresh orange maize hybrids with husk for higher PVA retention is determined by hybrid and harvesting time. There was decrease in %TRT of PVA as the harvesting time increases for roasted maize hybrids with husk.

### Effect of roasting method and harvesting time on sensory characteristics

Table5 showed the ANOVA results for the sensory properties of roasted orange maize evaluated at two locations and two seasons. The ANOVA results showed that hybrid, husk, and maturity had significant effects (*P* ≤ 0.001) on all the sensory properties, except husk that had no significant effect (*P* ≤ 0.05) on color and appearance. There was significant effect (*P* ≤ 0.05) of hybrids x maturity interaction on taste and overall acceptability. This observation suggested that hybrid, husk, and maturity affected the ratings of all the sensory properties of roasted orange maize and played a major role on the overall acceptability.

**Table 5:** Mean squares from the analysis of variance for the sensory properties of roasted orange maize evaluated at two locations for 2 years

Source	DF	MS					
		Color	Aroma	Chewiness	Taste	Appearance	Acceptability
Hybrid	7	27.20***	5.06*	49.50***	27.20***	6.16**	32.10***
Husk	1	0.09	14.10**	41.70***	89.70***	2.40	11.90***
Harvest time	2	58.80***	38.20***	376***	175***	24.60***	48.60***
Hybrid × husk	7	1.09	1.61	3.79	3.42	2.04	8.64**
Hybrid × harvest time	14	4.81**	2.66	5.07	5.07*	2.54	1.91
Husk × harvest time	2	0.53	8.30*	10.90*	9.25*	0.49	5.09**
Hybrid × husk × harvest time	125	2.15	1.79	3.33	2.86	1.61	8.22*
Error		2.04	1.97	3.16	2.83	1.94	2.37

*, **, ***Significant at *P* ≤ 0.05, *P* ≤ 0.01, and *P* ≤ 0.001, respectively. ns, not significant *P* > 0.05.

Tables6 and 7 showed the summary of descriptive statistics for sensory properties of roasted fresh orange maize hybrids with and without husk. For roasted fresh orange maize hybrids without husk, there was gradual increase in the rating of color and aroma at 20DAP and 27DAP before a decrease was observed at 34DAP. However, color showed no statistical mean difference across the three maturity stages. Aroma only showed no statistical difference at 20DAP and 27DAP but showed significant difference at 34DAP. It was observed that the chewiness, taste, and appearance showed a decrease across the harvest maturity stages. There were significant mean differences for chewiness and taste, while overall acceptability ratings showed no significant mean differences across the three maturity stages. The overall acceptability showed optimum rating at 20DAP (6.52) for roasted orange maize hybrid without husk and hybrid 1 was also found to have higher overall acceptability rating across harvest maturity stages.

**Table 6:** Descriptive statistics of sensory properties of roasted yellow hybrid maize without husk at different harvesting time at two locations for 2 years (*N* = 96)

Maturity		Color	Aroma	Chewiness	Taste	Appearance	Acceptability
20DAP	Mean	6.17a	6.04a	6.13a	6.39a	6.46a	6.52a
	Min	5.45	5.75	5.55	5.85	6.15	6.30
	Max	6.45	6.50	6.55	6.90	6.75	6.80
	LSD (0.05)	0.318	0.290	0.401	0.369	0.275	0.794
	SE	0.040	0.029	0.041	0.043	0.030	0.021
	CV (%)	0.641	0.482	0.664	0.668	0.470	0.320
27DAP	Mean	6.40a	6.09a	5.13b	5.84b	6.35a	6.13a
	Min	5.85	5.70	4.65	5.20	5.90	5.65
	Max	6.70	6.85	5.90	6.70	6.75	6.85
	LSD (0.05)	0.318	0.290	0.401	0.369	0.275	0.794
	SE	0.039	0.046	0.060	0.056	0.030	0.046
	CV (%)	0.613	0.752	1.17	0.964	0.473	0.748
34DAP	Mean	6.12a	5.51b	4.29c	5.28c	6.03b	6.14a
	Min	5.50	5.15	3.65	4.15	5.85	5.50
	Max	6.40	5.75	4.85	5.80	6.50	8.40
	LSD (0.05)	0.318	0.290	0.401	0.369	0.275	0.794
	SE	0.036	0.028	0.044	0.068	0.028	0.116
	CV (%)	0.590	0.509	1.03	1.29	0.467	1.88

Values with similar letters in column do not differ significantly (*P* < 0.05). DAP, days after pollination; LSD, least significant difference.

**Table 7:** Descriptive statistics of sensory properties of roasted yellow hybrid maize with husk at different harvesting time at two locations for 2 years (*N* = 96)

Maturity		Color	Aroma	Chewiness	Taste	Appearance	Acceptability
20DAP	Mean	6.21a	6.21a	5.87a	6.31a	6.51a	6.44a
	Min	5.15	5.50	5.00	5.55	5.95	5.90
	Max	6.85	6.60	6.70	7.00	7.00	6.80
	LSD (0.05)	0.314	0.323	0.384	0.379	0.278	0.347
	SE	0.081	0.043	0.060	0.052	0.047	0.034
	CV (%)	1.30	0.690	1.03	0.816	0.721	0.531
27DAP	Mean	6.11a	5.54b	4.76b	5.39b	6.21b	5.61b
	Min	5.25	4.95	3.95	4.35	6.00	4.85
	Max	6.60	6.50	6.60	6.95	6.60	6.70
	LSD (0.05)	0.314	0.323	0.384	0.379	0.278	0.347
	SE	0.062	0.058	0.103	0.093	0.026	0.068
	CV (%)	1.01	1.05	2.17	1.72	0.412	1.22
34DAP	Mean	5.89a	5.21c	3.89c	4.74c	5.82c	5.24c
	Min	4.95	4.90	3.35	4.10	5.55	4.75
	Max	6.25	5.60	4.30	5.55	6.05	5.60
	LSD (0.05)	0.314	0.323	0.384	0.379	0.278	0.347
	SE	0.055	0.026	0.042	0.051	0.022	0.035
	CV (%)	0.934	0.503	1.07	1.08	0.372	0.673

Values with similar letters in column do not differ significantly (*P* < 0.05). DAP, days after pollination; LSD, least significant difference.

However, the data on sensory properties for roasted orange maize hybrid with husk showed a general decrease in the mean ratings for all sensory properties. Aroma, chewiness, taste, appearance, and acceptability showed significant differences across the maturity stages except for color (Table7). The harvesting time had an effect on the ratings for all sensory properties except color that had no effect. The color, chewiness, and taste ratings showed similar patterns when compared with those of roasted orange maize hybrid without husk. The overall acceptability rating was not similar to that of roasted fresh orange maize hybrid without husk. This observation suggested that maturity and husk had an effect on the likeness of roasted maize with husk and roasted maize without husk, respectively.

## Discussion

The results of our studies revealed that hybrids, harvesting time, and husk had major effects on the concentrations of *β*-carotene and PVA values of the maize hybrids investigated. These results were in accordance with Menkir et al. (2008) that reported significant effects of inbred line and location on concentrations of provitamin A carotenoids (*P* ≤ 0.001) for tropical-adapted orange maize inbred lines. It could be concluded that harvesting time affects the *β*-carotene and PVA values because of the observed general increase in *β*-carotene and PVA values as harvesting time increases. This could be due to an increase in accumulation of dry matter and decrease in moisture content as the maize kernel matured. As solids accumulate, moisture declines from about 70% to ˜30% at maturity, which occurs at about 50–60 days after pollination (Hilson and Penny 1965).

When fresh cobs of orange maize hybrids were roasted without husk, both hybrid 7 at 20DAP and hybrid 5 at 27DAP, and 34DAP had the highest concentration of *β*-carotene and PVA value and suggesting that these genotypes retained more *β*-carotene and PVA at a specific harvesting time. Hybrid 5 at 34DAP emerged as the best with the highest values of *β*-carotene and PVA. The *β*-carotene and PVA concentrations of cobs roasted with husk were higher than those of the unprocessed but the unprocessed were higher than those of the roasted ones without husk. The *β*-carotene concentrations and PVA values of roasted fresh orange maize hybrids with husk were higher than the values for roasted fresh orange maize hybrids without husk. This suggested a positive effect of husk on the roasted fresh orange maize hybrids. The presence of husk could be an insulator to the maize cob when being roasted with hot charcoal and reduced the degradation of the PVACs. Hybrid 5 was the best when fresh orange maize hybrids were roasted without husk and hybrid 7 was the best when they were roasted with husk 34DAP and was the best harvesting time for higher *β*-carotene and PVA concentration for both hybrids and for the two forms of roasting. Studies with human subjects that were fed with cooked high—carotene biofortified maize showed the average conversion factor of yellow maize B-carotene to retinol was reported to be 3.2–1 (Muzhingi et al. 2011). The US Recommended Dietary Allowance of vitamin A for nearly all reference men, women, and children are 900, 700, and 400 *μ*g retinol activity equivalents, respectively (Food and Nutrition Board of the Institute of Medicine 2001). Here a 100 g portion of roasted maize (at 34DAP) hybrids 5 and 7 with 64 *μ*g and 827 *μ*g of PVA equivalents, respectively can provide 202 and 258 *μ*g of retinol. This shows that biofortified orange maize is a good source of vitamin A in humans.

Husk had an effect on the *cis* to *trans*- *β*-carotene ratio when fresh cobs of orange maize hybrids were roasted because the roasted maize hybrids with husk had a lower ratio than the roasted maize hybrids without husk at the three harvesting time. The lowest ratio was observed at 34DAP as found for roasted maize hybrids with husk and suggesting that 34DAP is the best harvesting time to roast maize hybrids in order to have low isomerization of *β*-carotene from *cis* to *trans* isomers.

The %TRT (TBC) and %TRT of PVA for roasted fresh orange maize hybrids with husk were generally higher than that of roasted fresh orange maize hybrids without husk. The best method to roast fresh orange maize hybrids is with husk and at 34DAP. Hybrid 5 was found to have better %TRT (TBC) at 27DAP irrespective of the roasting method. Higher %TRT (TBC) observed in this study was in agreement with those reported in the literature and it has been reported that cooking can increase the extractability and bioavailability of carotenoids (Dietz et al. 1988; Hart and Scott 1995). Howard et al. (1999) also reported that microwave cooking increased tissue degradation and increased the amount of carotenoids available for extraction. This observation probably reflects the fact that carotenoids in plants are sequestered into protein complexes and that cooking helped to release them (Baumann et al. 1982; Grimme and Brown 1984). However, the optimum acceptability rating was observed at 20DAP for orange hybrid maize roasted with and without husk, but the acceptability rating for roasted orange hybrid maize without husk was higher than that of roasted fresh yellow hybrid maize with husk.

## Conclusions

There was a general increase in *β*-carotene and PVA values as the harvesting time increases. The best time to harvest and consume roasted maize hybrid was found to be 34DAP and must be roasted with the husk. However, the consumers preferred roasted maize without husk at 20DAP. The major sensory properties that determined the acceptance/likeness of roasted fresh orange maize hybrids were found to be aroma, chewiness, and taste. Hybrid 5 was the best when fresh orange maize hybrid cobs were roasted without husk and hybrid 7 was the best when they were roasted with husk. This study supports the feasibility of maize biofortification as a promising intervention to combat VAD in developing countries, especially Nigeria.

## References

[b1] Ayatse JO, Eka OU, Ifon ET (1983). Chemical evaluation of the effect of roasting and nutritive value of maize, *zea mays* Linn. Food Chem.

[b2] Barampama Z, Simard RE (1995). Effect of soaking cooking and fermentation in composition, in-vitro starch digestibility and nutritive value of common beans. Plant Foods Hum. Nutr.

[b3] Baumann T, Weber G, Grimme LH (1982). Carotenoid and chlorophyll compositions of light harvesting and reaction centre proteins of the thylakoid membrane. Photobiochem. Photobiophys.

[b4] Dietz JM, Sri KS, Erdman JW (1988). Reversed phased HPLC analysis of *α*-carotene and *β*-carotene from selected raw and cooked vegetables. Plants Foods Hum. Nutr.

[b5] Egesel CO, Wang JC, Lambert RJ, Rocheford TR (2003). Combining ability of maize inbred for carotenoids and tocopherols. Crop Sci.

[b6] Egesel CO, Wong JC, Lambert RJ, Rocheford TR (2004). Gene dosage effects on carotenoid concentration in maize grain. Maydica.

[b7] FAOSTAT (2011). http://www.faostat.fao.org.

[b8] Food and Nutrition Board of the Institute of Medicine (2001). Chapter 4—vitamin A of dietary reference intakes for vitamin A, vitamin K, arsenic, boron, chromium, copper, iodine, iron, manganese, molybdenum, nickel, silicon, vanadium, and zinc.

[b9] Gibson RS, Hotz ZC (2001). Dietary diversification/modification strategies to enhance micronutrient content and bioavailability of diets in developing countries. Br. J. Nutr.

[b10] Grimme LH, Brown JS (1984). Functions of chlorophylls and carotenoids in thylakoid membranes. Adv. Photosyn Res.

[b11] Hart DJ, Scott KJ (1995). Development and evaluation of an HPLC method for the analysis of carotenoids in foods, and the measurement of the carotenoids content of vegetables and fruits commonly consumed in UK. Food Chem.

[b12] Hilson MT, Penny LH (1965). Dry matter accumulation and moisture loss during maturation of corn grain. Agron. J.

[b13] Howard LA, Wong AD, Perry AK, Klein BP (1999). *β*-carotene and ascorbic acid retention in fresh and processed vegetables. J. Food Sci.

[b14] Howe JA, Tanumihardjo SA (2006). Evaluation of analytical methods for carotenoid extraction from biofortified maize (Zea mays sp). J. Agric. Food Chem.

[b15] Jones G, Steketee RW, Black RE, Bhutta ZA, Morris SS, Bellagio Child Survival Study Group (2003). How many child deaths can we prevent this year?. Lancet.

[b16] Khachik F, Goli MB, Beecher GR, Holden J, Lusby WR, Tenorio MD (1992). Effects of food preparation on quantitative distribution of major carotenoid constituents of tomatoes and several vegetables. J. Agric. Food Chem.

[b17] Kurilich AC, Juvik AJ (1999). Quantification of carotenoid and tocopherol antioxidants in zea mays. J. Agric. Food Chem.

[b18] Larmond E (1977). Laboratory methods for foods.

[b19] Lonzano-Alejo N, Vasquez Carrillo G, Pixley K, Rojas NP (2007). Physical properties and carotenoid content of maize kernels and its mixtamalized snacks. Innovative Food Sci. Emerg. Technol.

[b20] Menkir A, Maziya-Dixon B (2004). Influence of genotype and environment of *β*-carotene content of tropical yellow endosperm maize genotypes. Maydica.

[b21] Menkir A, Liu W, White WS, Maziya-Dixon B, Rocheford T (2008). Carotenoids diversity in tropical-adapted orange maize inbred lines. Food Chem.

[b22] Mosha TC, Pace RD, Adeyeye S, Laswai HS, Mtebe K (1997). Effect of traditional processing practices on the content of total carotenoids, *β*-carotene, *α*-carotene and vitamin A activity of selected Tanzanian vegetables. J. Plant Foods Hum. Nutr.

[b23] Murphy EW, Criner PE, Gray BC (1975). Comparison of methods for calculating retention nutrients in cooked foods. J. Agric. Food Chem.

[b24] Muzhingi T, Gadaga HT, Siwela HA, Grusak MA, Russel RM, Tang G (2011). Yellow maize with high *β*-carotene is an effective source of vitamin A in healthy Zimbabwean men. Am. J. Clin. Nutr.

[b25] Okoh PN, Osagie AU, Eka OU (1998). Nutritional quality of plant foods. ISBN 9782120022.

[b26] Osanyintola OJ, Marek JH, Akingbala JO (1992). Effect of time of harvest and variety on the quality of boiled green field maize (*Zea Mays Linn*. Trop. Sci.

[b27] Ruel MT (2001).

[b28] SAS Institute (2000). SAS guide for personal computers.

[b29] Watts BM, Ylimaki GL, Jeffery LSE (1989). Basic sensory methods for food evaluation.

[b30] Weber EJ (1987). Carotenoids and tocols of corn grain determined by HPLC. J. Am. Oil Chem. Soc.

[b31] West KP (2003). Vitamin A deficiency disorders in children and women. Food Nutr. Bull.

